# Transcriptomic insights into citrus segment membrane’s cell wall components relating to fruit sensory texture

**DOI:** 10.1186/s12864-018-4669-y

**Published:** 2018-04-23

**Authors:** Xun Wang, Lijin Lin, Yi Tang, Hui Xia, Xiancong Zhang, Maolan Yue, Xia Qiu, Ke Xu, Zhihui Wang

**Affiliations:** 1 Institution of Pomology & Olericulture, Sichuan Agricultural University, No 211 Huimin Road, Wenjiang District, Chengdu, 611130 Sichuan China; 2 Sichuan Horticultural Crop Agrotechnical Promotion Workstation, No 4 Wuhou Memorial Temple Street, Wuhou District, Chengdu, 610041 Sichuan China; 3 College of Horticulture, Sichuan Agricultural University, No 211 Huimin Road, Wenjiang District, Chengdu, 611130 Sichuan China

**Keywords:** Cell wall components, Citrus, Fruit develop, RNA-seq, Segment membrane, Sensory texture

## Abstract

**Background:**

During fresh fruit consumption, sensory texture is one factor that affects the organoleptic qualities. Chemical components of plant cell walls, including pectin, cellulose, hemicellulose and lignin, play central roles in determining the textural qualities. To explore the genes and regulatory pathways involved in fresh citrus’ perceived sensory texture, we performed mRNA-seq analyses of the segment membranes of two citrus cultivars, Shiranui and Kiyomi, with different organoleptic textures.

**Results:**

Segment membranes were sampled at two developmental stages of citrus fruit, the beginning and end of the expansion period. More than 3000 differentially expressed genes were identified. The gene ontology analysis revealed that more categories were significantly enriched in ‘Shiranui’ than in ‘Kiyomi’ at both developmental stages. In total, 108 significantly enriched pathways were obtained, with most belonging to metabolism. A detailed transcriptomic analysis revealed potential critical genes involved in the metabolism of cell wall structures, for example, *GAUT4* in pectin synthesis, *CESA1*, *3* and *6*, and *SUS4* in cellulose synthesis, *CSLC5*, *XXT1* and *XXT2* in hemicellulose synthesis, and *CSE* in lignin synthesis. Low levels, or no expression, of genes involved in cellulose and hemicellulose, such as *CESA4, CESA7*, *CESA8*, *IRX9* and *IRX14*, confirmed that secondary cell walls were negligible or absent in citrus segment membranes. A chemical component analysis of the segment membranes from mature fruit revealed that the pectin, cellulose and lignin contents, and the segment membrane’s weight (% of segment) were greater in ‘Kiyomi’.

**Conclusion:**

Organoleptic quality of citrus is easily overlooked. It is mainly determined by sensory texture perceived in citrus segment membrane properties. We performed mRNA-seq analyses of citrus segment membranes to explore the genes and regulatory pathways involved in fresh citrus’ perceived sensory texture. Transcriptomic data showed high repeatability between two independent biological replicates. The expression levels of genes involved in cell wall structure metabolism, including pectin, cellulose, hemicellulose and lignin, were investigated. Meanwhile, chemical component contents of the segment membranes from mature fruit were analyzed. This study provided detailed transcriptional regulatory profiles of different organoleptic citrus qualities and integrated insights into the mechanisms affecting citrus’ sensory texture.

**Electronic supplementary material:**

The online version of this article (10.1186/s12864-018-4669-y) contains supplementary material, which is available to authorized users.

## Background

Fruit quality is determined by many attributes. The organoleptic properties are the vital quality attributes that influence consumer appeal [1]. Among the organoleptic properties, texture is a major factor [2] that governs more than 30% of food palatability [3]. The sensory texture of food has been divided mechanically into several parameters, such as hardness, cohesiveness, brittleness and chewiness [4]. In fruit and vegetables, crispy and crunchy textures are particularly qualities desired by consumers because they contribute to enjoyment [5].

Plant cell walls play a central role in determining the textural quality of plant-based foods [6]. Fruit texture is primarily derived from the composition of cell walls and the middle lamella [7]. The main chemical components of plant cell walls and the middle lamella include pectin, cellulose, hemicellulose and lignin. The relative composition of the wall varies in different species, organs, tissues and cells, and is associated with plant growth, development, maturation and senescence. Edible plant tissues are usually rich in parenchymatous cells that contain mostly primary cell walls [8, 9]. In the primary wall, the cellulose consists of β-1,4-linked glucose, with a lower degree of polymerization, between 2000 and 6000 units, rather than the over 10,000 units in secondary cell walls. Cellulose imparts rigidity and tearing resistance to edible tissues [9]. The cell walls contain two other groups of branched polysaccharides, the pectins and hemicelluloses. Pectic polysaccharides are rich in galacturonic acid, and additionally contain rhamnose, arabinose and galactose in significant amounts. Pectins are also the major components of the middle lamella. Hemicelluloses are a diverse group of polysaccharides, in which xyloglucans are the prominent hemicelluloses in the primary walls of the edible vegetables and fruit of dicotyledonous plants [6]. Pectins and hemicelluloses confer plasticity and the ability to stretch [9]. Lignin is absent, or in very low concentrations, in the cells of fruit pulp and vegetables at their edible stages [6, 9]. Lignin affects the rigidity and cohesion of the walls [6].

Citrus fruit are hesperidia. The fruit are divided internally into 9–14 juice vesicle-containing segments by segment membranes [10]. Fresh citrus (mainly oranges and tangerines) consumption of both the flesh and segment membrane is predominant in many countries, including China, Mexico, India, Argentina and Brazil. Segment membranes are edible but non-target elements that have diverse properties among species. Generally, the segment membranes of orange are thin and with a low occurrence in the fibered pith. The neighboring segment membranes adhere tightly and are difficult to separate. In contrast, the segment membranes of tangerine are thicker and occur more often in the pith. The neighboring segment membranes are relatively easy to separate. Segment membrane properties significantly affect the organoleptic qualities of fresh citrus, making the fruit flimsy, crispy and easy to crumble, or harsh, viscous, gummy and difficult to crumble.

In recent years, citrus hybrid cultivar production has rapidly expanded in China with the focus on consumer-desired properties, such as juiciness, sweet and seedless flesh, and easy peeling. *Citrus unshiu* Marcov × *Citrus sinensis* Osbeck (Kiyomi tangor) and *Citrus reticulata* Blanco ‘Shiranui’ are two citrus hybrid cultivars that are rapidly increasing in popularity. ‘Kiyomi’ is an orange-mandarin cross, while ‘Shiranui’ is a three-way hybrid between ‘Kiyomi’ and *C. reticulata* Blanco ‘Ponkan’ [11]. The two cultivars are both easy to peel, sweet, juicy and seedless. However, the ‘Kiyomi’ fruit’s texture is disagreeable. Its segment membranes are thick, glutinous and chewy. Thus, domestic consumers tend to peel off segment membranes with their hands and eat the flesh only. In contrast, ‘Shiranui’ fruit has a more agreeable texture. We hypothesize that the organoleptic differences in the two cultivars’ segment membranes are associated with the composition of cell walls and the middle lamella. However, little is understood about this association and the underlying molecular mechanisms.

Here, to explore the molecular mechanisms of segment membrane’ development, we performed an RNA-seq analysis. Segment membranes were sampled at two developmental stages. The genes in the biosynthesis and regulatory pathways involved in determining the composition of cell walls and the middle lamella were emphasized. To clarify the putative association between cell wall composition and the differences in the two cultivars’ organoleptic properties, the major components in the mature fruit’s segment membranes were analyzed. The transcriptome analysis of segment membrane development combined with cell wall composition analyses should enhance our understanding of the effects of segment membranes on citrus organoleptic qualities.

## Methods

### Plant materials and RNA preparation

Five-year-old ‘Kiyomi’ tangor and ‘Shiranui’ trees cultivated in a citrus research orchard located in Suining, Sichuan Province, China, were used. Fruit samples were harvested at two developmental stages, 150 and 210 days after flowering, assigned as first and second stages, respectively. At the first stage, fruit development occurred during the early expansion period, while at the second stage, fruit expansion had finished, and fruit reached their maximum size and began to mature. For each cultivar, fruit samples were collected from three different trees. Thus, each tree was an independent biological replicate. For RNA-seq, two independent randomly chosen biological replicates were used. Ten representative fruit from each tree were collected at each sampling stage. Segment membranes separated from fruit segments were immediately frozen in liquid nitrogen and kept at − 80 °C until use. Total RNA was extracted using an RNAprep Pure Plant Kit (TIANGEN, Beijing, China), which was specifically designed for materials rich in polysaccharides and polyphenolics. Extracted RNA was treated with DNase (TIANGEN, Beijing, China) to remove genomic DNA. RNA quality was surveyed by separation in 1.0% agarose gels, stained with ethidium bromide and observed by UV light.

### RNA-seq library construction and sequencing

Total RNA samples from two trees of each cultivar at two developmental stages, for a total of eight samples, were used for RNA-seq library construction and sequencing. Sequencing was performed on an Illumina HiSeq 2000 platform. RNA quantity and integrity were determined using a Nanodrop 2000 (Thermo Scientific, Waltham, MA, USA) and a Bioanalyzer 2100 (Agilent Technologies, Santa Clara, CA, USA). RNA-seq libraries were constructed using the Illumina TruSeq RNA Library Prep Kit v2 (Illumina, San Diego, CA, USA) following standard protocols. Poly(A)-containing transcripts were enriched from the total RNA using oligo(dT)-coated magnetic beads. For each sample, adapters with unique barcodes were ligated to the end-polished cDNA fragments. The libraries were amplified by PCR and quantitated by Qubit 2.0 (Thermo Scientific).

### Gene annotation, expression level comparison and differential expression analysis

Kallisto [12] was used for quantification with default parameters. Each repeat group had a very high correlation coefficient (*r* > 0.97), and differentially expressed genes (DEGs) were identified from each comparison using edgeR [13]. DEGs were annotated with the Pfam database [14]. Gene Ontology (GO) enrichment was performed by TopGO [15], and KOBAS [16] was applied for the Kyoto Encyclopedia of Genes and Genomes [17] enrichment. Other data analyses and visualization were performed by self R scripts.

### Quantitative RT-PCR analysis

First-strand cDNA was synthesized from total RNA using an anchored-oligo (dT)_18_ primer with the Transcriptor First-Strand cDNA Synthesis Kit with gDNA Eraser (Aidlab, China). The Applied Biosystems StepOnePlus™ Real-Time PCR System equipped with StepOne™ Software V2.1 (Bio-Rad, USA) was used. The β-actin expression level was used for normalization. Water was the negative control. Each reaction was replicated three times. To confirm the specific amplification of desired PCR products, melting curves were analyzed. The qRT-PCR data were analyzed using the comparative ΔΔC_T_ method [18]. All of the reactions were performed with three biological replicates.

### Segment membrane’ weight-ratio determination

The weight ratios of segment membranes to whole segments of mature fruits were determined. For each cultivar, the three trees sampled for RNA-seq and qRT-PCR were used. The data from each tree were regarded as a replicate. Ten fruits were sampled from each tree. Fruit segments were separated and weighed. After carefully removing juice pulp from inside the segment, the segment membranes were weighed. The segment membrane’ weight ratio was given as the value (%) of the segment membrane to fruit segment.

### Pectin content and pectin methylation analysis

The segment membranes used for weight-ratio determination were dried and ground to a 60-mesh powder. The powder was used for pectin content and pectin methylation analyses, and the following experiments, which included determinations of the lignin, cellulose and hemicellulose contents. The data from each tree were regarded as a replicate. Pectin content was determined by quantifying GalA using the carbazole colorimetric method [19]. The extent of pectin methylesterification was determined by the quantification of the released methanol [20, 21].

### Analyses of lignin, cellulose and hemicellulose contents

The lignin content was the sum of the Klason insoluble and soluble lignin. Klason insoluble lignin was analyzed according to the Tappi T 222 om-88 as described by Bura et al. [22]. Soluble lignin in hydrolysate from the Klason analysis was monitored using a UV spectrophotometer at 205 nm. Cellulose and hemicellulose contents were calculated from glucose and xylose contents, respectively, in the Klason analysis’ hydrolysate. The glucose and xylose contents were determined using high-performance liquid chromatography (Flexar, PerkinElmer, Inc., Waltham, MA, USA) with a refractive index detector. The lactose (0.5 g/L, Sigma, USA) was used as an internal standard. Each item’s measurement was repeated three times.

## Results and discussion

### Summary of the transcriptomic sequencing dataset and gene differential expression levels

**Fig. 1:**
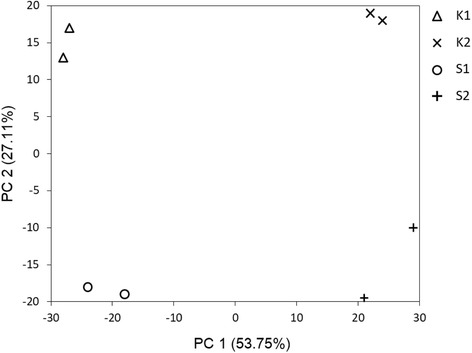
Principal component analysis of gene expression. K1: ‘Kiyomi’ at first stage; K2: ‘Kiyomi’ at second stage; S1: ‘Shiranui’ at first stage; S2: ‘Shiranui’ at second stage

Total RNAs from eight samples (two citrus cultivars sampled at two developmental stages were performed two biological replicates) were prepared and sequenced on an Illumina HiSeq 2000 platform. The output was an average 10 M reads (Paired-end 100-bp model) for each library. High repeatability was shown between two independent biological replicates. From a principal component analysis (Fig. 1), the eight samples were clearly separated in the first principal component (PC)1 × PC2 score plots. The total variable of PC1 reached to 53.75%, and PC1 was clearly separated between two developmental stages. The total variable of PC2 was 27.11% and was separated between the two cultivars.

All transcriptomes and unigenes were realigned. Read counts were normalized by transcripts per million, and the R package’s edgeR was used to call DEGs. The DEG cut-offs were a false discovery rate (FDR) < 0.005 and an absolute log_2_ fold change > 1. A DEG-based heatmap was supplied in Additional file 1: Figure S1. More than 3000 DEGs were identified. The numbers of DEGs in the four comparisons were listed in Table 1.

**Table 1:** Number of differentially expressed genes

Sample comparisons	Number of different expression genes
K1 vs. S1	575
K2 vs. S2	649
K1 vs. K2	1578
S1 vs. S2	1205

K1 and K2: ‘Kiyomi’ samples from first and second developmental stages, respectively; S1 and S2: ‘Shiranui’ samples from first and second developmental stages, respectively

Eight DEGs were used in a qRT-PCR assay to further validate the transcriptome sequencing dataset. Primers used in quantitative RT-PCR experiments were listed in Additional file 1: Table S1. The comparison of the qRT-PCT analysis to RNA-seq expression levels was provided in Additional file 1: Figure S2. Positive correlations were exhibited in the four sample comparisons, showing R^2^ to be 0.5413 (‘Kiyomi’ first-stage vs. ‘Shiranui’ first-stage), 0.7469 (‘Kiyomi’ second-stage vs. ‘Shiranui’ second-stage), 0.5313 (‘Kiyomi’ first-stage vs. ‘Kiyomi’ second-stage), and 0.7893 (‘Shiranui’ first-stage vs. ‘Shiranui’ second-stage).

### Determination of biological processes and pathway enrichment

The GO enrichment analysis identified 76 GO categories as significantly enriched at the first stage, including 67 categories in ‘Shiranui’ and 33 in ‘Kiyomi’, with 24 categories that overlapped. At the second stage, in agreement with the biological processes majority in ‘Shiranui’ at the first stage, 72 categories were identified to be significantly enriched in ‘Shiranui’ and 42 in ‘Kiyomi’, with 28 categories overlapped, and totally 86 GO categories were significantly enriched at the second stage. In comparisons between the different developmental stages of each cultivar, the GO-enrichment analysis identified 131 biological processes as significantly enriched in ‘Kiyomi’, with 93 processes at both the first and second stages, and 55 overlapping processes. In ‘Shiranui’, 112 processes were enriched, 77 at the first stage and 80 at the second stage, with 45 overlapping processes.

**Fig. 2:**
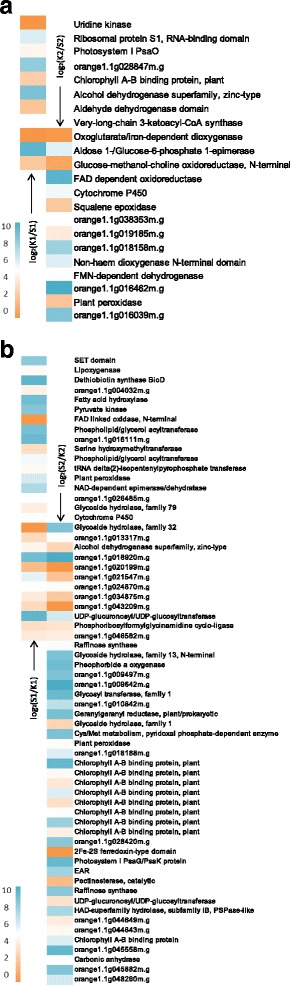
Up-regulated genes in KEGG metabolism category in the comparison of ‘Kiyomi’ to ‘Shiranui’ (**a**) and in the comparison of ‘Shiranui’ to ‘Kiyomi’ (**b**). The orange-to-blue scale represents a decreasing log_2_-fold change of gene expression. K1/S1: the comparison of ‘Kiyomi’ to ‘Shiranui’ at the first stage; K2/S2: the comparison of ‘Kiyomi’ to ‘Shiranui’ at the second stage; S1/K1: the comparison of ‘Shiranui’ to ‘Kiyomi’ at the first stage; S2/K2: the comparison of ‘Shiranui’ to ‘Kiyomi’ at the second stage

A total of 108 significantly enriched pathways were obtained. At the first stage, 67 and 36 pathways were enriched in ‘Shiranui’ and ‘Kiyomi’, respectively. Similarly, at the second-stage, 63 and 42 pathways were enriched in ‘Shiranui’ and ‘Kiyomi’, respectively. The top 30 significantly enriched pathways in ‘Kiyomi’ or ‘Shiranui’ were shown in Additional file 1: Figure S3. At the first stage, the pathways “glycolysis/gluconeogenesis”, “pyrimidine metabolism” and “plant hormone signal transduction” were the most significantly enriched in ‘Kiyomi’. In ‘Shiranui’, all of the pathways indicated similar and relatively low levels of enrichment. At the second stage, “peroxisome” was highly significantly enriched in ‘Kiyomi’, and “photosynthesis-antenna proteins”, “plant-pathogen interaction” and “circadian rhythm-plant” were significantly enriched in ‘Shiranui’. Pathways were grouped according to KEGG pathway maps (http://www.genome.jp/kegg/pathway.html). Most pathway groups belonged to the metabolism category, including “carbohydrate metabolism”, “energy metabolism”, “lipid metabolism”, “amino acid metabolism”, “metabolism of cofactors and vitamins” and “metabolism of terpenoids and polyketides” (Additional file 1: Figures S3–S6). DEGs in the metabolism category were presented in Fig. 2.

Comparing the different developmental stages, 74 and 72 enriched pathways were identified at the first and second stages, respectively, in ‘Kiyomi’, while in ‘Shinarui’, 67 enriched pathways were identified at both the first and second stages. The top 30 significantly enriched pathways at the first and second stages were shown in Additional file 1: Table S2.

The cell wall bestows rigidity to the plant and texture to plant tissues, and the cellular components are possibly responsible for the taste and texture of citrus segment membranes. In growing plants, the major cell wall components are cellulose, hemicellulose, pectin, lignin and a small amount of structural proteins. Cellulose, hemicellulose and pectin are carbohydrates, which impart cell-wall rigidity, increase tensile strength and provide the ability to resist compression. Lignin, a polymer of aromatic alcohols, is hard and renders considerable strength to the cell wall. Enriched pathway results reinforced these conclusions. For example, in the carbohydrate metabolism group, which was frequently enriched, “starch and sucrose metabolism”, “amino sugar and nucleotide sugar metabolism” and “pentose phosphate pathway”, were key pathways responsible for the synthesis and degradation of pectin, cellulose and hemicellulose. Accordingly, we investigated the expression levels of genes involved in the metabolism of cell wall structures.

### Pectin metabolism-related transcriptomic insights

Pectins are a group of polysaccharides predominantly composed of GalA. Homogalacturonan (HG), the homopolymer of GalA (D-GalA) linked in an α-1,4 configuration, is the most abundant pectic polysaccharide. HG biosynthesis is catalyzed by α-1,4-D-galacturonosyltransferase (EC 2.4.1.43), designated GAUT, and some GAUT-like (GATL) proteins [23]. Twelve *GAUTs*, *GAUT1–15*, with the exception of *GAUT2*, *5* and *13*, were detected in our research. *GAUT4*, *8*, *9* and *15* showed slightly higher expression levels than other *GAUTs*, with the average expression levels from the two citrus cultivars at both developmental stages being greater than 10 (Additional file 1: Table S3). No differential expression of the four genes was found between ‘Kiyomi’ and ‘Shiranui’ at either the first or second stage, with the exceptions that *GAUT4* and *GAUT8* were expressed significantly higher (*p* < 0.05) in the second stage than in the first stage in both cultivars. *GAUT1* and *GAUT7* showed similar and relatively low expression levels (Additional file 1: Table S3). *Arabidopsis GAUT4-* and *GAUT1*-encoding proteins were confirmed as crucial to plant growth and development, and their mutations may be lethal [23]. Here, citrus *GAUT4* and *GAUT1* also showed detectable levels, indicating these potentially crucial roles. *GAUT4* was expressed more highly among the *GAUT* genes, suggesting its important role in citrus. The similar expressing patterns of *GAUT1* and *GAUT7* in our results might be explained by their functioning in a protein complex [24]. *GAUT2* was not detected in either ‘Kiyomi’ or ‘Shiranui’, which was in agreement with GAUT2 being a nonfunctional truncated homolog of GAUT1 [25]. Several GAUT-like genes were detected, including *GATL2–10*, with the exception of *GATL6* and *GATL8*. The expression levels of the *GATLs* were similar to those of the *GAUT* genes (Additional file 1: Table S3).

**Fig. 3:**
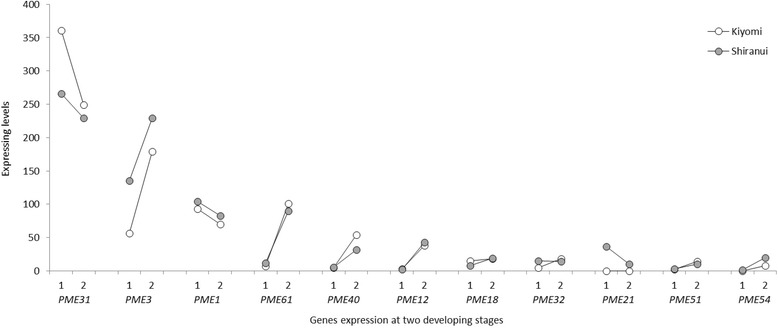
*PME* transcript expression levels in ‘Kiyomi’ and ‘Shiranui’ at two developmental stages. 1: at the first stage; 2: at the second stage. *PME* genes on the *X*-axis are listed in the order of the average expression levels from highest to lowest

There is a group of pectinases that broadly control pectin degradation. Pectin methylesterase (PME, E.C. 3.1.1.11) is an enzyme that catalyzes the specific hydrolysis of the methyl ester bond at C-6 in GalA residues in the linear HG domain of pectin, which is secreted in a highly (70%–80%) methylesterified form. In total, 23 integral *PME* isoform genes were detected in this research. Several *PME*s showed higher expression levels than the HG biosynthesis-related *GAUT* and *GATL* genes. For instance, the average *PME31* expression level from ‘Kiyomi’ and ‘Shiranui’ at the two developmental stages was 276.333, which was significantly higher than that of *GAUT8* (17.261) or *GATL3* (16.460). *PME* transcripts displaying expression levels greater than 10 were listed in Additional file 1: Table S3, and there were 11 transcripts in total. At the first stage, 8 of the 11 *PME*s showed higher expression levels in ‘Shiranui’ than in ‘Kiyomi’ (Fig. 3; Additional file 1: Table S3), but at the second stage, only 6 *PME*s had higher expression levels (Fig. 3; Additional file 1: Table S3). The *PME*s tended to be expressed higher in the second stage than in the first stage. In ‘Kiyomi’, 9 of 11 *PME*s had increased expression levels in the second stage (Fig. 3; Additional file 1: Table S3), while in ‘Shiranui’ 7 had increased expression levels in the second stage (Fig. 3; Additional file 1: Table S3). PMEs have diverse functions in plant growth, and their transcripts and protein activities are likely to involve the recruitment of pools of specific isoforms [26]. The roles of PMEs are linked to cell wall extension and stiffening, cellular separation, dry fruit dehiscence and soft fruit ripening [27]. The *PME*s detected in our research were predicted to be involved in at least two processes, cell wall extension and fruit ripening. The multiple roles of PMEs might explain why *PME*s were generally expressed to greater extents than the *GAUT*s and *GATL*s. A similar result was observed in a tomato (*Solanum lycopersicum*) study, in which gene expression was visually analyzed on ethidium bromide-stained gels [28].

A number of genes encoding PME inhibitor (PMEI) family proteins, which restrain PMEs through a post-translational control mechanism, were also detected in this research. There were nine *PMEI* contigs that had expression levels greater than 10 (Additional file 1: Table S3). The expression levels of *PMEI*s were comparative to, or even higher than, those of the *PME*s (Additional file 1: Table S3).

Seven genes encoding pectin acetylesterase (E.C. 3.1.1.6), which is responsible for removing the acetyl residues of pectin, were found in our research. The pectin acetylesterase 8, 2 and 6 genes had expression levels greater than 10 and were listed in Additional file 1: Table S3.

The deesterified pectin is subsequently degraded by pectinases that break the α-1,4-linkages. Pectinases function by hydrolysis, such as polygalacturonase (E.C. 3.2.1.15), or through transelimination mechanisms, such as pectate lyase (E.C. 4.2.2.2) and pectin lyase (E.C. 4.2.2.10). Here, 32 independent contigs belonging to the pectinase superfamily were detected, although the gene names of these contigs were not annotated by querying using a BLAST algorithm-based search against *Arabidopsis* protein databases. Twelve contigs, with expression levels greater than 10 in any sample, were shown in Additional file 1: Table S3. The levels of pectinase activity in the first and second stages might not have been as great as those of the GAUTs, GATLs and PMEs.

### Cellulose metabolism-related transcriptomic insights

**Fig. 4:**
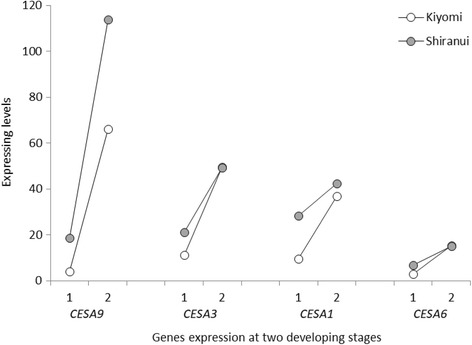
*CESA* transcript expression levels in ‘Kiyomi’ and ‘Shiranui’ at two developmental stages. 1: at the first stage; 2: at the second stage. *CESA* genes on the *X*-axis are listed in the order of the average expression levels from highest to lowest

Cellulose is a linear homopolymer of β-1,4-linked glucose residues in plant. Its synthesis is organized by specific plasma membrane-bound cellulose synthase (CESA; EC 2.4.1.12) complexes in symmetrical rosette forms [29]. We detected seven *CESA* genes, *CESA1*, *3*, *4*, *6*, *7*, *8* and *9*, that were expressed in citrus fruit segment membranes (Additional file 1: Table S4). Among them, *CESA4*, *7* and *8*, showed very low expression levels. The other four, *CESA1*, *3*, *6* and *9*, displayed relatively high expression levels, which all increased from the first to second stage (Fig. 4). At the first stage, the expression levels of the four genes in ‘Shiranui’ were all greater than in ‘Kiyomi’. At the second stage, *CESA9* maintained a significantly higher expression level in ‘Shiranui’. *CESA1* also had a higher expression level in ‘Shiranui’, but it was not statistically significant. *CESA3* and *6* had similar expression levels in the two cultivars. The *Arabidopsis* genome has at least 10 *CESA*s [30]. *Arabidopsis CESA4*, *7* and *8* genes combine to form the CESA rosette required for secondary wall synthesis [31–34]. The expression patterns of the three genes were highly correlated [35–37]. In our research, the co-expression of *CESA4*, *7* and *8* at low levels indicated week or negligible secondary cell-wall formation occurred in citrus segment membranes. In *Arabidopsis*, at least three of the four CESA proteins, CESA1, 2, 3 and 6, are required for primary wall synthesis. CESA1 and 3 appear to be absolutely required, whereas CESA2 and 6 may be at least partially redundant [37–41]. The absence of the *CESA2* gene in our results indicated the requirement for CESA1, 3 and 6 in citrus segment membranes’ primary wall synthesis. The lower level of *CESA6* relative to *CESA1* and *CESA3* inferred that the former is potentially redundant. Plant primary cell walls are generally thin and less rigid, whereas secondary cell walls are rigid and responsible for most of the plant’s mechanical support. In mature fruit’s flesh cells, primary cell walls are dominant, while secondary cell walls are virtually absent [6, 9, 42]. From the expression levels of *CESA*s in our research, it was confirmed that primary cell walls were dominant in citrus segment membranes.

**Fig. 5:**
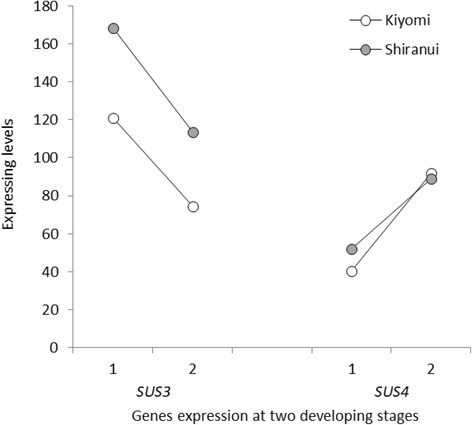
*SUS* transcript expression levels in ‘Kiyomi’ and ‘Shiranui’ at two developmental stages. 1: at the first stage; 2: at the second stage. *SUS* genes on the *X*-axis are listed in the order of the average expression levels from highest to lowest

Sucrose synthase (SUS, also SuSy; EC 2.4.1.13) is another important enzyme involved in cellulose synthesis. Four SUS-encoding genes were detected in our research. *SUS3* and *4* showed significantly high expression levels comparing with those of *SUS2* and *6* (Additional file 1: Table S4). The *SUS4* expression increased from the first to second stage in both ‘Kiyomi’ and ‘Shiranui’ (Fig. 5). It was expressed higher in ‘Shiranui’ than in ‘Kiyomi’ at the first stage, whereas at the second stage, there was no obvious difference in its expression level between the two citrus varieties. *SUS3* showed a higher expression level in ‘Shiranui’ than in ‘Kiyomi’ at both developmental stages. SUS is associated directly with the CESA complex and contributes to cellulose biosynthesis [43]. Here, citrus *SUS4* had a 91% amino acid identity with *Gossypium hirsutum* SuSy (GenBank U73588), which may be associated with cellulose synthesis [44]. In our transcriptome data, *SUS4* displayed an expression pattern similar to those of *CESA1*, *3* and *6*, which are associated with cellulose synthesis in primary walls. We hypothesized that SUS4 might be the main protein responsible for cellulose synthesis in citrus primary cell walls.

We detected three genes encoding cellulases (CEL; EC 3.2.1.4), including endo-β-1,4-glucanase. Compared with *CESA* and *SUS*, the expression levels of the *CEL*s were low (Additional file 1: Table S3). *CEL3* was the only one with a relatively high expression level, which increased from the first to second stage in both cultivars, and was expressed higher in ‘Shiranui’ at both stages. *CEL* genes were expressed at low levels in our experimental materials, which is in accordance with the opinion that plant cellulases actually endohydrolyze cellulose and are not strong enough to cause the bulk degradation of cellulose microfibrils [45].

### Hemicellulose metabolism-related transcriptomic insights

Hemicellulose is a diverse group of polysaccharides. It constitutes roughly one-third of the wall biomass. In dicots, the hemicelluloses are primarily xyloglucans (XyGs) and xylans (Xyls). The former is most abundant in the primary cell wall, and the latter in the secondary cell wall [46].

**Fig. 6:**
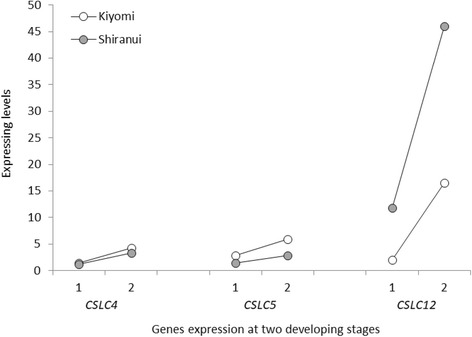
*CSLC* transcript expression levels in ‘Kiyomi’ and ‘Shiranui’ at two developmental stages. 1: at the first stage; 2: at the second stage. *CSLC* genes on the *X*-axis are listed in the order of the average expression levels from highest to lowest

XyGs have a β-1,4-glucan backbone that is linked with different mono-, di- or trisaccharide side-chain xylosyl residues. XyG biosynthesis requires glucan synthase to form the glucan backbone and glycosyl transferases to produce the side chains. At least five types of enzymatic activities are involved in XyG biosynthesis: cellulose synthase-like C proteins (CSLCs), XyG xylosyltransferases (XXTs), galactosyltransferase, fucosyltransferase and acetyltransferase. All of the genes encoding these enzymes were expressed in this study (Additional file 1: Table S5). The CSLCs and XXTs are responsible for the synthesis of the glucan backbone and xylosyl residues, respectively, which are the two major components of XyGs. Three *CSLC* genes, *CSLC4*, *5* and *12*, were detected in citrus segment membranes. *CSLC4* and *5* showed similar but lower expression levels than *CSLC12* (Fig. 6). The *Arabidopsis CSLC4* functions in the homocomplex CSLC4–CSLC4 and heterocomplex CSLC4–XXT [46]. It is highly expressed in all *Arabidopsis* tissues, and, within a tissue, its expression was in keeping with the expression levels of *XXT*s and corresponded with the tissue’s developmental timing [47, 48]. Here, however, *CSLC4* had a relatively low expression level, which might imply a less significant role in XyG synthesis in citrus segment membranes. CSLC5 is another important enzyme involved in the synthesis of the glucan backbone of XyG [49], but it is limited to specific tissues [47]. The similar levels of *CSLC4* and *5* in our results implied complementary roles in citrus segment membranes. Another *CSLC* gene, *CSLC12*, showed a relatively higher expression level than either *CSLC4* or *CSLC5*. Limited reports addressing CSLC12 implied that it is a potential XyG-synthesis candidate. *CSLC12* was expressed in all of the tested *Arabidopsis* organs, including roots, stems, flowers and seedlings [50]. In addition, like the *Arabidopsis* CSLC12 protein, citrus CSLC12 showed high homology levels to rice CSLC 1 (identity 72%), CSLC 7 (identity 73%) and CSLC 9 (identity 74%), which are expressed during different stages of ovule development [51].

**Fig. 7:**
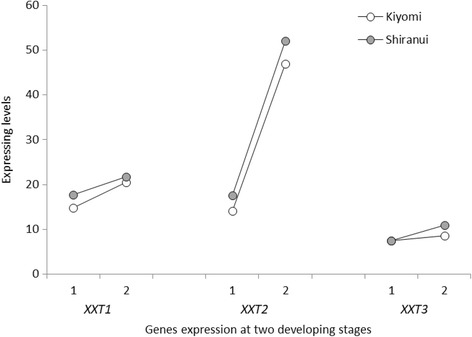
*XXT* transcript expression levels in ‘Kiyomi’ and ‘Shiranui’ at two developmental stages. 1: at the first stage; 2: at the second stage. *XXT* genes on the *X*-axis are listed in the order of average expression levels from highest to lowest

Three *XXT* genes, *XXT1*, *2* and *3*, were found in the present materials. The expression levels of *XXT1* and *XXT2* were higher in all of the materials than the expression levels of *XXT3* (Fig. 7). Five XXTs (XXT1–5) have been confirmed in *Arabidopsis* [52]. Among the five proteins, XXT1, 2 and 5 were confirmed to form hetero- or homo-complexes, such as XXT1–XXT2, XXT2–XXT2 and XXT2–XXT5 [46]. Additionally, in the major *Arabidopsis* tissues, *XXT2* has an approximately twofold greater expression level than *XXT1* and *5* [47]. In our data, at the second stage, XXT2 showed a twofold higher expression level than XXT1, but at the first stage its expression level was similar to that of XXT1. The connections in the XXT1–XXT2 and XXT2–XXT2 complexes may be temporal. *XXT3*, but not *XXT5*, was detected in our study. XXT5 is not as essential a protein for XyG formation as XXT1 and XXT2 [52], but the lack of this protein dramatically impacts XyG biosynthesis (Zabotina, 2012). In the *Arabidopsis xxt5* mutant, the absence of the XXT5 protein may be compensated for by other xylosyltransferases [53]. The citrus XXT5 function may have been compensated for by XXT1, 2 or 3. A XyG xylosyltransferase activity for XXT3 is suggested in *Arabidopsis* [54], which supports our hypothesis that XXT3 plays a compensating role for XXT5 in citrus.

Xyl is the major hemicellulose in the secondary walls of dicots [55], which is composed of a linear β-1,4-linked D-xylosyl backbone with various side branches [56]. The glycosyltransferases, irregular xylem phenotype (IRX) 9, 10, 14, 9-like (−L), 14-L, 10-L, 7/fragile fiber 8, fragile fiber 8 homolog and IRX8/GAUT12, appeared to be essential for Xyl backbone synthesis, the addition of side chains and the synthesis of the reducing-end structures [56–58]. Among these proteins, IRX9, 14 and fragile fiber 8 homologs were not detected in our research (Additional file 1: Table S5). *IRX9* and *14* were the only genes confirmed experimentally to have XylT activity [59–61]. *IRX9-L*, *14-L* and *10-L* act as partially redundant genes [55], although they are close homologs to *IRX9*, *14* and *10*, respectively. The inability to detect *IRX9* and *14* in our research suggested a low level of XylT activity and limited Xyl contents in citrus segment membranes. Because Xyl is the major hemicellulose in secondary cell walls of dicots [55], the limited Xyl content indicated weak or negligible secondary cell-wall formation. This was in accordance with the cellulose biosynthesis in which low levels of *CESA4*, *7* and *8*, being responsible for cellulose synthesis in secondary cell walls, were detected.

### Lignin metabolism-related transcriptomic insights

Lignins are complex natural polymers that result from the oxidative coupling of 4-hydroxyphenylpropanoids [62]. The synthesis of monolignols from phenylalanine requires deamination, hydroxylation, methylation and two successive reductions [63].

**Fig. 8:**
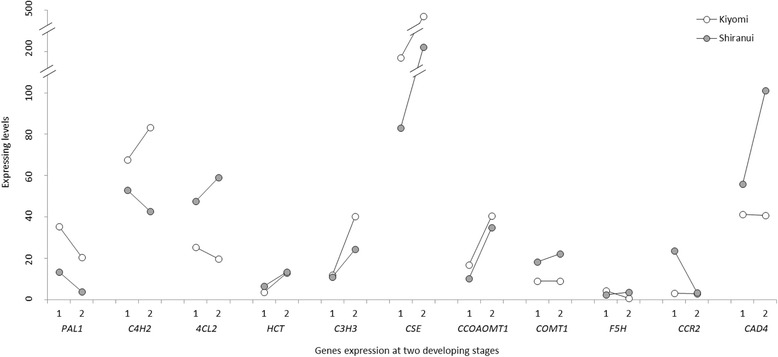
Expression levels of transcripts involved in lignin biosynthesis in ‘Kiyomi’ and ‘Shiranui’ at two developmental stages. 1: at the first stage; 2: at the second stage. Genes on the *X*-axis are listed in the order of the lignin biosynthesis pathway

The synthesis of caffeoyl-CoA from phenylalanine represents a subset of the upstream steps of the lignin biosynthetic pathway, particularly coniferyl alcohol (G lignin) and sinapyl alcohol (S lignin) [63]. Six enzymes were involved in this subset, phenylalanine ammonia lyase (PAL), cinnamate 4-hydroxylase (C4H), 4-coumarate:CoA ligase (4CL), *p*-coumaroyl shikimate 3-hydroxylase (C3H), hydroxycinnamoyl CoA:shikimate hydroxycinnamoyl transferase (HCT) and caffeoyl shikimate esterase (CSE). In our data, *PAL1*, *C4H2*, *4CL2*, *C3H3*, *HCT* (single gene) and *CSE* (single gene) were detected at considerable levels. After *CSE*, *C4H2* was the highest expressed gene. Its average expression level in all eight samples was 61.683, which was greater than *PAL1* (18.372), *4CL2* (38.071), *HCT* (9.259) and *C3H3* (21.952) (Fig. 8). In *Populus trichocarpa*, C4H2 associates with C3H3 and C4H1 to form heterodimeric (C4H1–C4H2, C4H1–C3H3 or C4H2–C3H3) and heterotrimeric (C4H1–C4H2–C3H3) membrane protein complexes that catalyze three converts, from cinnamic acid to *p*-coumaric acid, from *p*-coumaric acid to caffeic acid, and from *p*-coumaroyl shikimic acid to caffeoyl shikimic acid [64]. Two C4H genes, *C4H1* and *C4H2*, were confirmed in citrus. *C4H2* was constitutively expressed at a significantly higher level than *C4H1*, which is expressed in response to wound induction, even in wounded tissues [65]. From our data, the expression level of *C4H2* was extremely greater than the 0.111 *C4H1* level, which implied that the putative heterodimeric complex of C4H2–C3H3 was preferred, rather than the complexes containing C4H1. In addition, the high level of *C4H2* indicated that C4H2 might function in both the C4H2–C3H3 complex and in C4H2 alone. The CSE activity, combined with that of 4CL, produces caffeoyl-CoA from caffeoyl shikimate, bypassing the HCT reaction that occurs in the direct conversion [66]. *CSE*s were highly expressed in our data, with an average value of 245.820. The high expression indicated that the CSE pathway was more effective than that of HCT, which had been suggested in *Arabidopsis* [66]. However, considering the significantly higher expression of *CSE* compared with other genes, it is important to try and identify additional CSE functions.

Cinnamoyl CoA reductase (CCR) and cinnamyl alcohol dehydrogenase (CAD) are responsible for the final two steps of the lignin monomer synthesis pathway. Two *Arabidopsis CCR*s were cloned [67, 68]. *AtCCR1* is preferentially expressed in tissues undergoing lignification, while, in contrast, *AtCCR2* is involved in defense responses. In our research, *CCR1* showed a negligibly low expression level, with an average of 0.107, which suggested the faint lignification of citrus segment membranes during the two developmental stages. Five *CAD*s, *CAD1*, *4* and *7*–*9*, were detected in the citrus segment membranes, and *CAD4* (64.898 level) was more highly expressed than the others. Because the knockdown of *CAD4* in *Arabidopsis* resulted in plants with relatively normal lignin contents [69], we did not believe that the higher *CAD4* expression level would lead directly to a higher lignin content.

In addition to *HCT*, *CSE* and *F5H* (ferulate 5-hydroxylase), other genes involved in monolignol synthesis were detected to be members of multi-gene families. The highest expressing member of each gene family was compared among citrus samples (Fig. 8). Most genes including *HCT*, *C3H3*, *CCoAOMT1* (caffeoyl CoA *O*-methyltransferase) and *COMT1* (caffeic acid *O*-methyltransferase), had higher expression levels at the second stage compared with the first stage in both ‘Kiyomi’ and ‘Shiranui’. The comparisons of gene expression levels in the two citrus cultivars at the same developmental stage showed that, *PAL1*, *C4H2*, *C3H3*, *CCOAOMT1* and *CSE* were expressed higher in ‘Kiyomi’ at both stages, while *4CL2*, *HCT*, *CCR2*, *CAD4* and *COMT1* were expressed higher in ‘Shiranui’. The series of enzymes participating in the lignin synthesis pathway represent multiple isoforms encoded by different genes. These genes vary in kinetic properties, distributions, expression patterns, and responses to antisense suppression by the different enzyme isoforms [63]. Therefore, it is not possible to determine the specific gene responsible for the final lignin deposition. Here, in rarely lignified developing citrus segment membranes, the initial catalysis steps, e.g., the reaction catalyzed by C4H2, might determine the level of lignin deposition and provide substrates for downstream reactions.

### Chemical component analysis in mature fruit-segment membranes

**Fig. 9:**
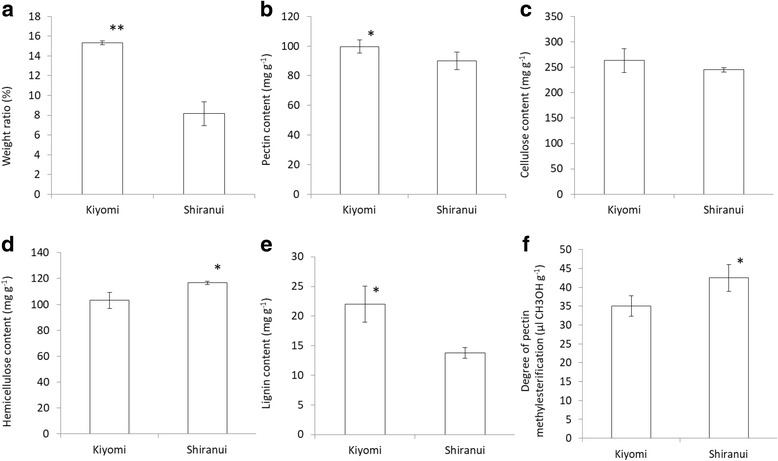
Component properties of segment membranes in ‘Kiyomi’ and ‘Shiranui’ mature fruit. **a** The weight (%) of segment membranes per segment; **b** Pectin content; **c** Cellulose content; **d** Hemicellulose content; **e** Lignin content; **f** Degree of pectin methylesterification. * indicated significant differences at *p* < 0.05 and ** indicated significant differences at *p *< 0.01 assessed by Student–Newman–Keuls test

The weight (%) of segment membranes per segment, and the main components contents of segment membrane’ cell walls, including pectin, cellulose, hemicellulos and lignin, and the degree of pectin methylesterification were illustrated in Fig. 9. The segment membrane weight of ‘Kiyomi’ was 15.32 ± 0.18%, which is significantly greater than that of ‘Shiranui’ at 8.16 ± 1.19% (*p* < 0.001). Among the main components of the cell wall, the pectin, cellulose and lignin contents were all greater in ‘Kiyomi’, especially the pectin and lignin contents, which showed significantly higher levels than in ‘Shiranui’. The hemicellulose, unlike the other components, was more abundant in ‘Shiranui’ at 116.6 ± 1.43 mg g^− 1^ than in ‘Kiyomi’ at 103.2 ± 6.15 mg g^− 1^ (*p* < 0.05). The degree of pectin methylesterification was expressed as μl CH_3_OH g^− 1^. Based on the results (Fig. 9), ‘Shiranui’ was more methylesterified than ‘Kiyomi’.

Segment membranes are not targeted in breeding but are edible portions of citrus. The popular consumer-driven sensory properties of citrus segment membranes are thin, soft and brittle. The property of thinness increases the enjoyment of eating the juice sacs, which are the contents of the segments, rather than the segment membrane. Desirable brittleness in citrus segment membranes allows them to be easily crumbled while chewing the juice sacs. The softness of segment membranes is preferred to a harsh texture.

The segment membrane’ weight of ‘Shiranui’ was suspected to determine its thinness, making ‘Shiranui’ more pulpous. Pectin in plant cell walls is part of the polysaccharide network, which is involved in the control of cell wall porosity and cell expansion [70], and it is the major adhesive material between cells [71]. Adding pectin was suggested to improve food cohesiveness and, consequently, hardness and chewiness increased [72]. The greater amount of pectin in ‘Kiyomi’ segment membranes was surmised to impart its chewiness and decreased brittleness. Less esterified pectic polymers might also result in the decreased brittleness of ‘Kiyomi’ segment membranes because de-esterified pectin is susceptible to depolymerization, resulting in the increased viscosity of fruit paste [9] and, to some degree, citrus segment membranes that are less easily crumbled.

Lignin imparts strength and rigidity to plant walls, which are highly resistant to both mechanical disruption and enzymatic degradation [63, 73]. In animal nutritional tests, lignin and cellulose are undesired or “antinutritive” components because of their negative impact on nutrient availability [74] and palatability [75]. The harshness of foliage (as food) increases as the lignin and cellulose contents increase. The harsh taste in ‘Kiyomi’ results from the higher contents of these two components, particularly lignin, in segment membranes. Hemicellulose contains a diverse group of polysaccharides in plant cell walls. It has a random, amorphous structure that imparts little strength and is mostly soluble in aqueous solutions [55]. Accordingly, a higher hemicellulose content would not lead to a harsher taste for ‘Shiranui’.

## Conclusions

Our study provided a comprehensive transcriptomic insight into the citrus segment membrane’s cell wall structures and metabolism, and indicated the possible contribution of segment membrane cell-wall composition to fresh citrus’ organoleptic properties. The high repeatability of transcriptomic data and the detection of DEGs in ‘Kiyomi’ and ‘Shiranui’ indicated distinct differences during segment membrane development between the two citrus cultivars. A detailed transcriptomic analysis revealed potential critical genes involved in the metabolism of cell wall structures, for example, *GAUT4* in pectin synthesis, *CESA1*, *3* and *6*, and *SUS4* in cellulose synthesis, *CSLC5*, *XXT1* and *XXT2* in hemicellulose synthesis, and *CSE* in lignin synthesis. Low levels, or no expression, of genes involved in cellulose and hemicellulose, such as *CESA4, CESA7*, *CESA8*, *IRX9* and *IRX14*, confirmed that secondary cell walls were negligible or absent in citrus segment membranes. A chemical component analysis in mature fruit segment membranes showed that higher pectin, cellulose and lignin contents might result in the undesired organoleptic properties of ‘Kiyomi’, which resulted in the fruit being perceived as thick, glutinous and chewy.

## Additional file

Additional file 1: **Figure S1.** Heatmap of differentially expressing genes. **Figure S2.** Verification of RNA-seq by qRT-PCT. **Figure S3.** Top 30 significantly enriched pathways based on up-expressed genes in ‘Kiyomi’ compared with in ‘Shiranui’ at the first-stage. **Figure S4.** Top 30 significantly enriched pathways based on up-expressed genes in ‘Shiranui’ compared with in ‘Kiyomi’ at the first-stage. **Figure S5.** Top 30 significantly enriched pathways based on up-expressed genes in ‘Kiyomi’ compared with in ‘Shiranui’ at the second-stage. **Figure S6.** Top 30 significantly enriched pathways based on up-expressed genes in ‘Shiranui’ compared with in ‘Kiyomi’ at the second-stage. **Table S1.** Primers used in quantitative qRT-PCR experiments. **Table S2.** Top 30 significantly enriched pathways based on up-regulated genes. **Table S3.** Genes involved in pectin metabolism. **Table S4.** Genes involved in cellulose metabolism. **Table S5.** Genes involved in hemicellulose (xyloglucans and xylan) metabolism. (PDF 808 kb)

## Abbreviations

4CL: 4-coumarate:CoA ligase
C3H: *p*-coumaroyl shikimate 3-hydroxylase
C4H: Cinnamate 4-hydroxylase
CAD: Cinnamyl alcohol dehydrogenase
CCoAOMT: Caffeoyl CoA *O*-methyltransferase
CCR: Cinnamoyl CoA reductase
CEL: Cellulase
CESA: Cellulose synthase
COMT: Caffeic acid *O*-methyltransferase
CSE: Caffeoyl shikimate esterase
CSLCs: Cellulose synthase-like C proteins
DEGs: Differentially expressed genes
F5H: Ferulate 5-hydroxylase
FDR: False discovery rate
GATL: Galacturonosyltransferase-like
GAUT: Galacturonosyltransferase
GO: Gene Ontology
HCT: Hydroxycinnamoyl CoA:shikimate hydroxycinnamoyl transferase
HG: Homogalacturonan
IRX: Irregular xylem phenotype
PAL: Phenylalanine ammonia lyase
PC: Principal component
PME: Pectin methylesterase
SUS: Sucrose synthase
XXTs: Xyloglucans xylosyltransferases
XyGs: Xyloglucans
Xyls: Xylans
