# Supplementary material for: Transcriptomic insights into citrus segment membrane’s cell wall components relating to fruit sensory texture
Source: BMC Genomics. 2018 Apr 23;19:280. doi: 10.1186/s12864-018-4669-y (PMC5914067; doi:10.1186/s12864-018-4669-y)
Supplement: Supplementary file 1 — Figure S1. Heatmap of differentially expressing genes. Figure S2. Verification of RNA-seq by qRT-PCT. Figure S3. Top 30 significantly enriched pathways based on up-expressed genes in ‘Kiyomi’ compared with in ‘Shiranui’ at the first-stage. Figure S4. Top 30 significantly enriched pathways based on up-expressed genes in ‘Shiranui’ compared with in ‘Kiyomi’ at the first-stage. Figure S5. Top 30 significantly enriched pathways based on up-expressed genes in ‘Kiyomi’ compared with in ‘Shiranui’ at the second-stage. Figure S6. Top 30 significantly enriched pathways based on up-expressed genes in ‘Shiranui’ compared with in ‘Kiyomi’ at the second-stage. Table S1. Primers used in quantitative qRT-PCR experiments. Table S2. Top 30 significantly enriched pathways based on up-regulated genes. Table S3. Genes involved in pectin metabolism. Table S4. Genes involved in cellulose metabolism. Table S5. Genes involved in hemicellulose (xyloglucans and xylan) metabolism. (PDF 808 kb) [file 12864_2018_4669_MOESM1_ESM.pdf]

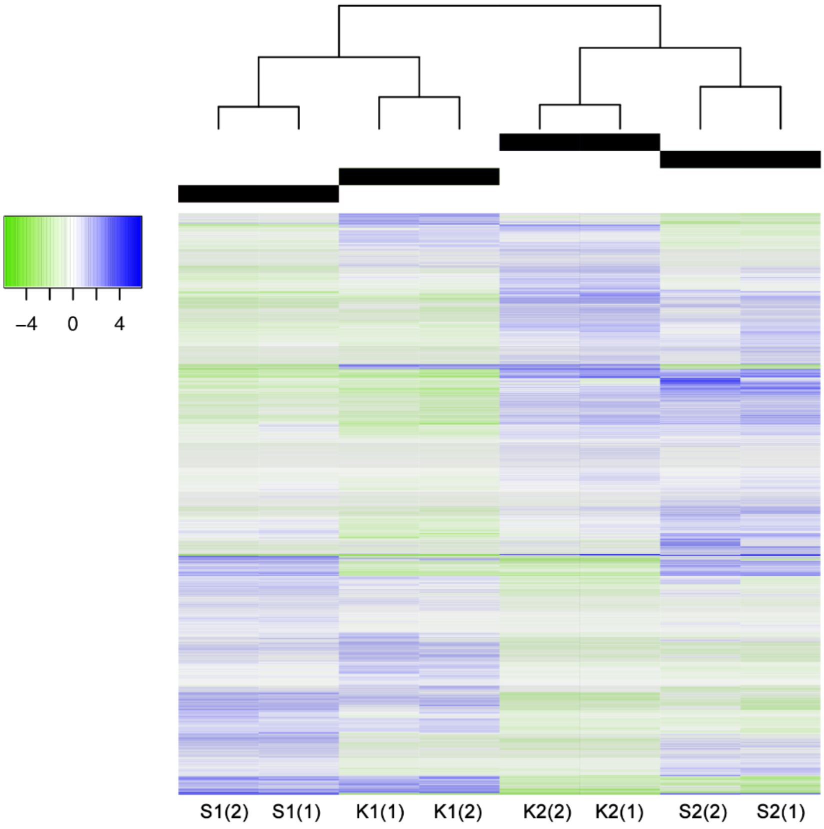


**Figure S1 Heatmap of differentially expressed genes.** The $\log_2$ value of the ratio between ‘Kiyomi’ and ‘Shiranui’ is presented. K1 and K2: ‘Kiyomi’ samples from first and second developmental stages, respectively; S1 and S2: ‘Shiranui’ samples from first and second developmental stages, respectively. The numbers in parentheses represent independent biological replicates.

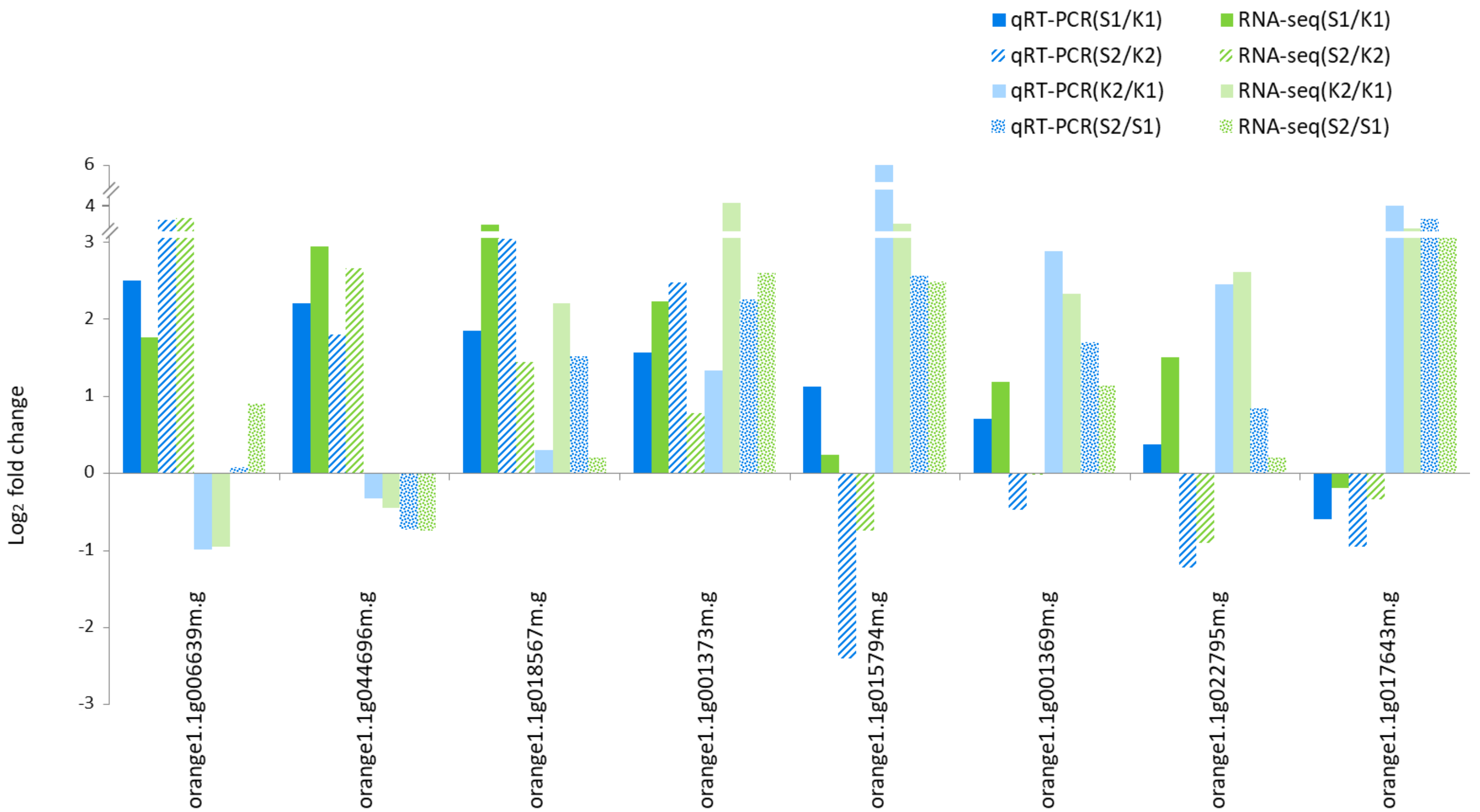


**Figure S2 Verification of RNA-seq by qRT-PCT.** S1/K1: Comparison of 'Shiranui' (first-stage) to 'Kiyomi' (first-stage); S2/K2: Comparison of 'Shiranui' (second-stage) to 'Kiyomi' (second-stage); K2/K1: Comparison of 'Kiyomi' (second-stage) to 'Kiyomi' (first-stage); S2/S1: Comparison of 'Shiranui' (second-stage) to 'Shiranui' (first-stage)

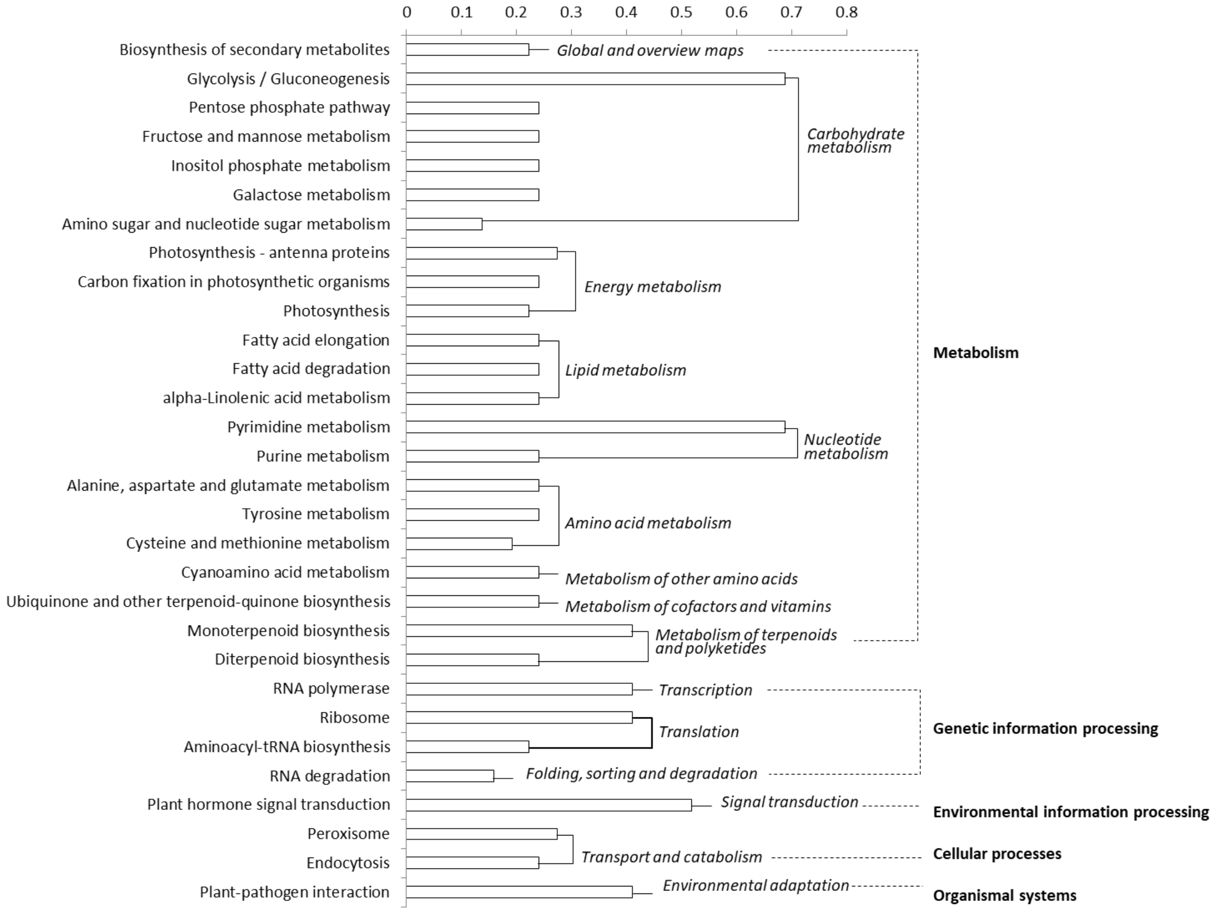


**Figure S3 Top 30 significantly enriched pathways based on up-expressed genes in 'Kiyomi' compared with in 'Shiranui' at the first-stage.** The pathways are grouped using solid lines according to http://www.genome.jp/kegg/pathway.html (group names are in italics). The groups are further classed into KEGG pathway maps using dotted lines (KEGG pathway map names are in bold). The pathway enrichment analysis was significant at FDR < 0.005. The *x*-axis represents the –log(FDR value).

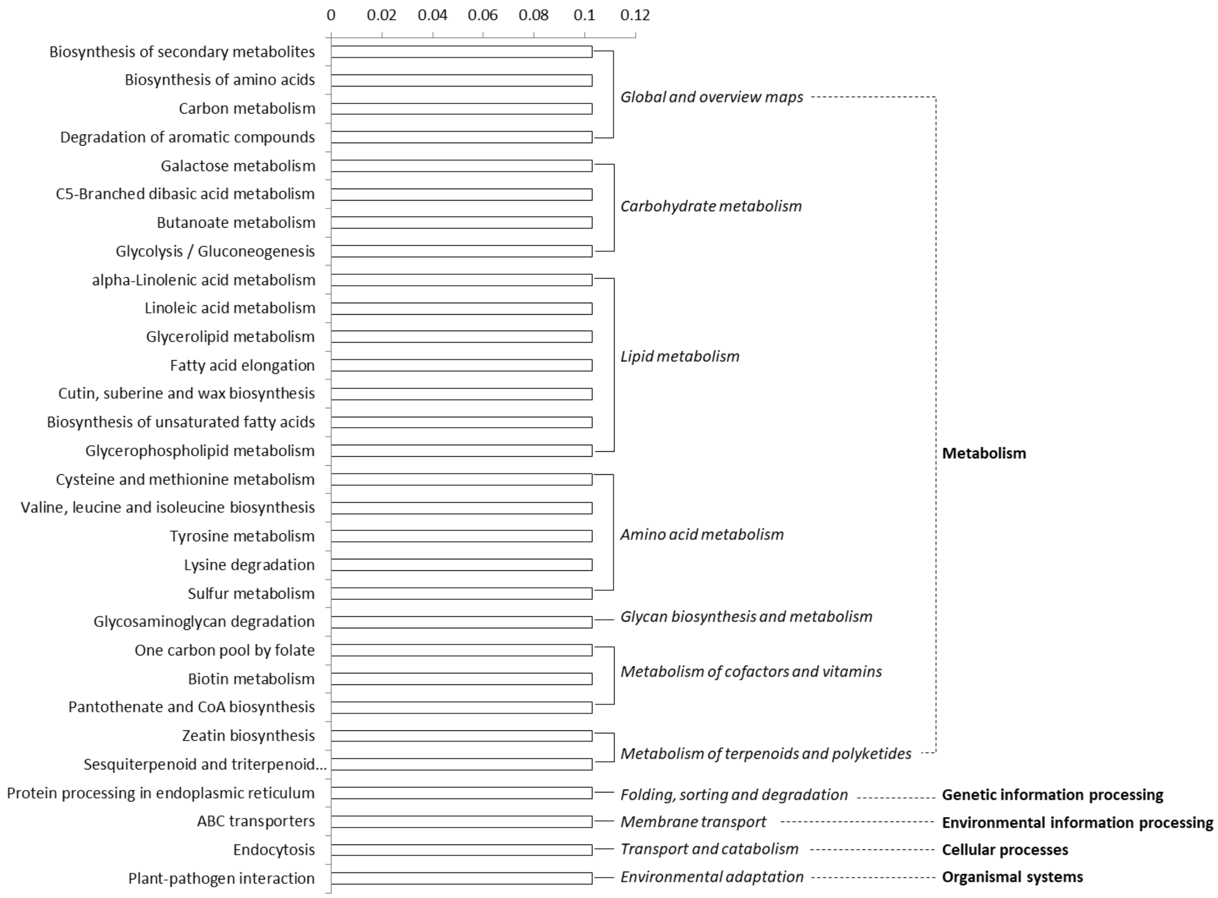


**Figure S4 Top 30 significantly enriched pathways based on up-expressed genes in 'Shiranui' compared with in 'Kiyomi' at the first-stage.** The pathways are grouped using solid lines according to http://www.genome.jp/kegg/pathway.html (group names are in italics). The groups are further classed into KEGG pathway maps using dotted lines (KEGG pathway map names are in bold). The pathway enrichment analysis was significant at FDR < 0.005. The *x*-axis represents the –log(FDR value).

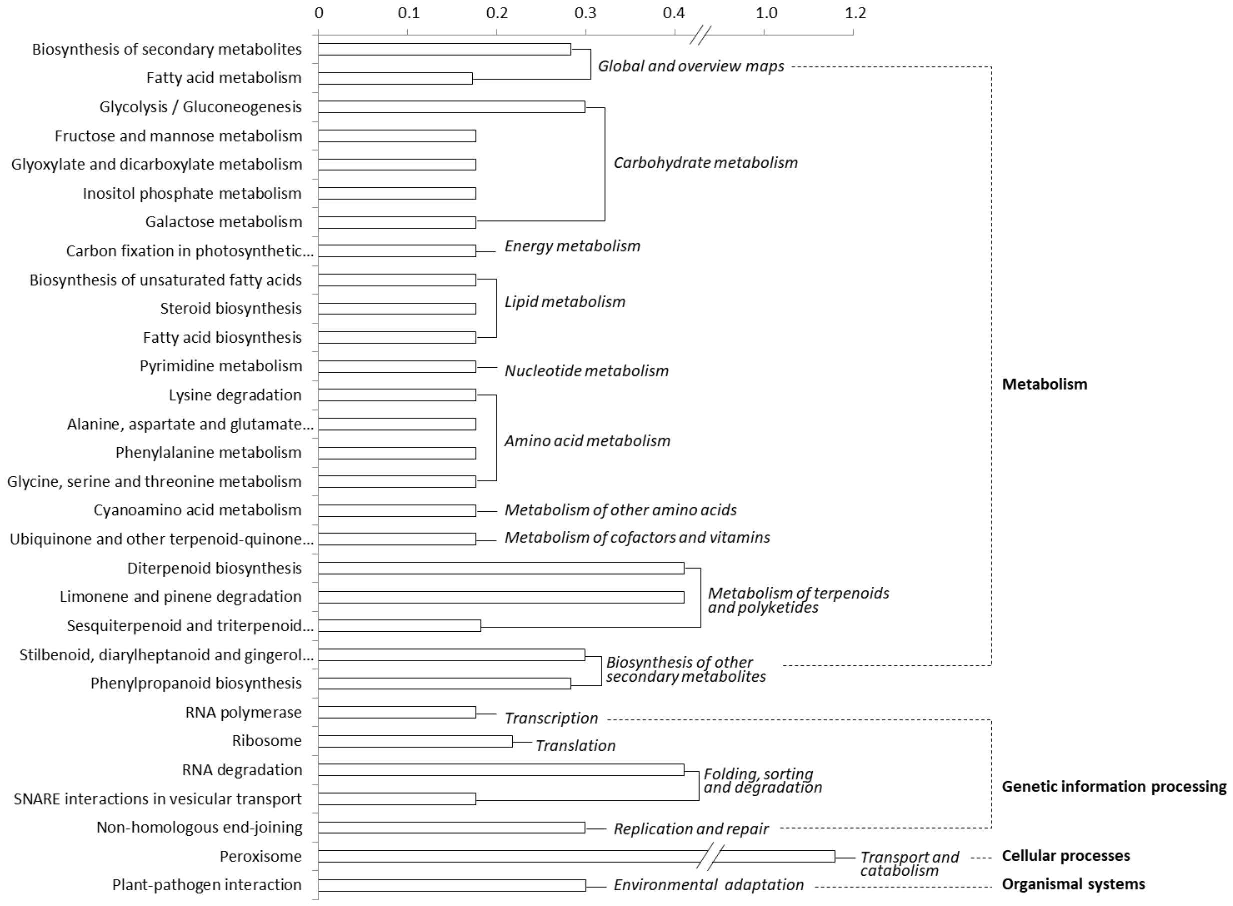


**Figure S5 Top 30 significantly enriched pathways based on up-expressed genes in 'Kiyomi' compared with in 'Shiranui' at the second-stage.** The pathways are grouped using solid lines according to http://www.genome.jp/kegg/pathway.html (group names are in italics). The groups are further classed into KEGG pathway maps using dotted lines (KEGG pathway map names are in bold). The pathway enrichment analysis was significant at FDR < 0.005. The *x*-axis represents the –log(FDR value).

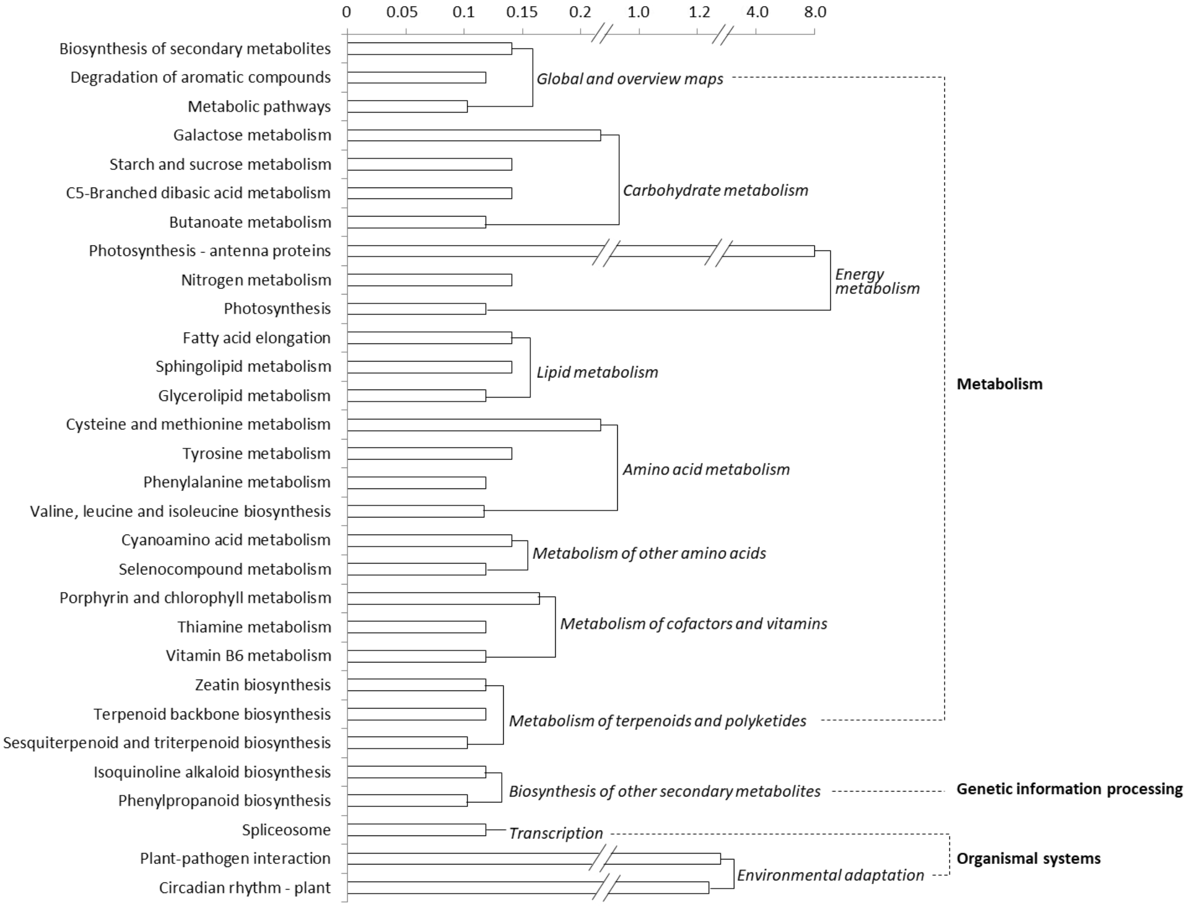


**Figure S6 Top 30 significantly enriched pathways based on up-expressed genes in 'Shiranui' compared with in 'Kiyomi' at the second-stage.** The pathways are grouped using solid lines according to http://www.genome.jp/kegg/pathway.html (group names are in italics). The groups are further classed into KEGG pathway maps using dotted lines (KEGG pathway map names are in bold). The pathway enrichment analysis was significant at FDR < 0.005. The *x*-axis represents the –log(FDR value).

**Table S1 Primers used in quantitative qRT-PCR experiments**

| Contigs | InterPro description | Primers (5'-3') |
|---|---|---|
| orange1.1g006639m.g | cellulose synthase-like B3 | Former: GTGGATCCTTACTTGGGGCT<br>Reverse: GCAGGAAAGTGGAGTTGGTG |
| orange1.1g044696m.g | 6-phosphogluconate dehydrogenase family protein | Former: CCCTTTACAGCATCCCTCCA<br>Reverse: ATCAGATTGTGGTGGGAGCA |
| orange1.1g018567m.g | galacturonosyltransferase-like 2 | Former: TGAAGAAGGTTGTTTGCGTCTT<br>Reverse: CATTCACTCCGTCCTACAACAC |
| orange1.1g001373m.g | cellulose synthase A9 | Former: AGTGGACGGGGATGAAGAAG<br>Reverse: AATGTGGTGCGGGTCTTTTC |
| orange1.1g015794m.g | Plant invertase/pectin methylesterase inhibitor superfamily | Former: GGGCTTATTTTGAGAACGTGGA<br>Reverse: TTCACTACTGTTTTGCCGATCC |
| orange1.1g001369m.g | cellulose synthase 6 | Former: TTGTGTACCCTTGGACCTCC<br>Reverse: TCCCAGTGAGAAGGCAGAAG |
| orange1.1g022795m.g | cellulose synthase-like D6 | Former: CGCTTGATTATCCGCCAGAG<br>Reverse: TGCAAACTTCCAAGCTTCCC |
| orange1.1g017643m.g | galacturonosyltransferase-like 7 | Former: TTAGATCCTCACACCTCGACAG<br>Reverse: GGAATACGGAGGCTTTTCGAAA |

InterPro descriptions are based on the *Arabidopsis thaliana* database.

**Table S2 Top 30 significantly enriched pathways based on up-regulated genes**

| Based on up-expressed genes in | Enriched pathways | –log(FDR) | Groups within KEGG pathway maps | KEGG pathway maps |
|---|---|---|---|---|
| K1 (K1 vs. K2) | Fatty acid metabolism | 0.4705 | Global and overview maps | Metabolism |
| | Inositol phosphate metabolism | 0.1286 | Carbohydrate metabolism | Metabolism |
| | Galactose metabolism | 0.1286 | Carbohydrate metabolism | Metabolism |
| | Glycolysis / Gluconeogenesis | 0.1230 | Carbohydrate metabolism | Metabolism |
| | Photosynthesis - antenna proteins | 0.2982 | Energy metabolism | Metabolism |
| | Nitrogen metabolism | 0.1732 | Energy metabolism | Metabolism |
| | Biosynthesis of unsaturated fatty acids | 0.6036 | Lipid metabolism | Metabolism |
| | Ether lipid metabolism | 0.5475 | Lipid metabolism | Metabolism |
| | Fatty acid biosynthesis | 0.5475 | Lipid metabolism | Metabolism |
| | Arachidonic acid metabolism | 0.3654 | Lipid metabolism | Metabolism |
| | Cutin, suberine and wax biosynthesis | 0.1914 | Lipid metabolism | Metabolism |
| | Cysteine and methionine metabolism | 0.4705 | Amino acid metabolism | Metabolism |
| | Valine, leucine and isoleucine biosynthesis | 0.2934 | Amino acid metabolism | Metabolism |
| | Tyrosine metabolism | 0.5475 | Amino acid metabolism | Metabolism |
| | Phenylalanine metabolism | 0.1914 | Amino acid metabolism | Metabolism |
| | Arginine and proline metabolism | 0.1914 | Amino acid metabolism | Metabolism |
| | Glutathione metabolism | 0.5475 | Metabolism of other amino acids | Metabolism |
| | Glycosphingolipid biosynthesis - ganglio series | 0.1914 | Glycan biosynthesis and metabolism | Metabolism |
| | Glycosaminoglycan degradation | 0.1230 | Glycan biosynthesis and metabolism | Metabolism |
| | Pantothenate and CoA biosynthesis | 0.1914 | Metabolism of cofactors and vitamins | Metabolism |

| | | | | |
|---|---|---|---|---|
| | Thiamine metabolism | 0.1286 | Metabolism of cofactors and vitamins | Metabolism |
| | Ubiquinone and other terpenoid-quinone biosynthesis | 0.1286 | Metabolism of cofactors and vitamins | Metabolism |
| | Vitamin B6 metabolism | 0.1230 | Metabolism of cofactors and vitamins | Metabolism |
| | Tropane, piperidine and pyridine alkaloid biosynthesis | 0.7342 | Biosynthesis of other secondary metabolites | Metabolism |
| | Isoquinoline alkaloid biosynthesis | 0.5475 | Biosynthesis of other secondary metabolites | Metabolism |
| | Protein processing in endoplasmic reticulum | 3.2613 | Folding, sorting and degradation | Genetic information processing |
| | Plant hormone signal transduction | 0.2982 | Signal transduction | Environmental information processing |
| | Endocytosis | 0.2497 | Transport and catabolism | Cellular processes |
| | Circadian rhythm - plant | 2.5470 | Environmental adaptation | Organismal systems |
| | Plant-pathogen interaction | 0.1978 | Environmental adaptation | Organismal systems |
| K2 (K1 vs. K2) | Biosynthesis of secondary metabolites | 0.8153 | Global and overview maps | Metabolism |
| | Fatty acid metabolism | 0.4995 | Global and overview maps | Metabolism |
| | Metabolic pathways | 0.4995 | Global and overview maps | Metabolism |
| | Inositol phosphate metabolism | 0.0634 | Carbohydrate metabolism | Metabolism |
| | Starch and sucrose metabolism | 0.0634 | Carbohydrate metabolism | Metabolism |
| | Amino sugar and nucleotide sugar metabolism | 0.0634 | Carbohydrate metabolism | Metabolism |
| | Ascorbate and aldarate metabolism | 0.0634 | Carbohydrate metabolism | Metabolism |
| | Pentose and glucuronate interconversions | 0.0634 | Carbohydrate metabolism | Metabolism |
| | Photosynthesis | 0.4989 | Energy metabolism | Metabolism |
| | Fatty acid elongation | 1.7468 | Lipid metabolism | Metabolism |
| | Cutin, suberine and wax biosynthesis | 0.6167 | Lipid metabolism | Metabolism |
| | Fatty acid biosynthesis | 0.4350 | Lipid metabolism | Metabolism |
| | Sphingolipid metabolism | 0.0634 | Lipid metabolism | Metabolism |
| | Biosynthesis of unsaturated fatty acids | 0.0634 | Lipid metabolism | Metabolism |
| | Steroid biosynthesis | 0.0634 | Lipid metabolism | Metabolism |
| | Linoleic acid metabolism | 0.0634 | Lipid metabolism | Metabolism |

| | | | | |
|---|---|---|---|---|
| | Fatty acid degradation | 0.0634 | Lipid metabolism | Metabolism |
| | Phenylalanine, tyrosine and tryptophan biosynthesis | 0.3555 | Amino acid metabolism | Metabolism |
| | Phenylalanine metabolism | 0.0634 | Amino acid metabolism | Metabolism |
| | beta-Alanine metabolism | 0.0634 | Metabolism of other amino acids | Metabolism |
| | Glycosaminoglycan degradation | 0.3555 | Glycan biosynthesis and metabolism | Metabolism |
| | Biotin metabolism | 0.0634 | Metabolism of cofactors and vitamins | Metabolism |
| | Porphyrin and chlorophyll metabolism | 0.0634 | Metabolism of cofactors and vitamins | Metabolism |
| | Sesquiterpenoid and triterpenoid biosynthesis | 0.4995 | Metabolism of terpenoids and polyketides | Metabolism |
| | Brassinosteroid biosynthesis | 0.3555 | Metabolism of terpenoids and polyketides | Metabolism |
| | Limonene and pinene degradation | 0.0634 | Metabolism of terpenoids and polyketides | Metabolism |
| | Phenylpropanoid biosynthesis | 1.7468 | Biosynthesis of other secondary metabolites | Metabolism |
| | Flavonoid biosynthesis | 0.0634 | Biosynthesis of other secondary metabolites | Metabolism |
| | Ribosome biogenesis in eukaryotes | 0.0634 | Translation | Genetic information processing |
| | ABC transporters | 0.0634 | Membrane transport | Environmental information processing |
| S1 (S1 vs. S2) | Fatty acid metabolism | 0.2452 | Global and overview maps | Metabolism |
| | Metabolic pathways | 0.0787 | Global and overview maps | Metabolism |
| | Biosynthesis of secondary metabolites | 0.0787 | Global and overview maps | Metabolism |
| | Butanoate metabolism | 0.0787 | Carbohydrate metabolism | Metabolism |
| | Glyoxylate and dicarboxylate metabolism | 0.2452 | Carbohydrate metabolism | Metabolism |
| | Inositol phosphate metabolism | 0.1191 | Carbohydrate metabolism | Metabolism |
| | Pentose and glucuronate interconversions | 0.0787 | Carbohydrate metabolism | Metabolism |
| | Starch and sucrose metabolism | 0.0787 | Carbohydrate metabolism | Metabolism |
| | Ether lipid metabolism | 0.8660 | Lipid metabolism | Metabolism |
| | Fatty acid biosynthesis | 0.3085 | Lipid metabolism | Metabolism |
| | Biosynthesis of unsaturated fatty acids | 0.2452 | Lipid metabolism | Metabolism |
| | Cutin, suberine and wax biosynthesis | 0.1694 | Lipid metabolism | Metabolism |

| | | | | |
|---|---|---|---|---|
| | Glycerophospholipid metabolism | 0.1694 | Lipid metabolism | Metabolism |
| | Cysteine and methionine metabolism | 0.2452 | Amino acid metabolism | Metabolism |
| | Valine, leucine and isoleucine biosynthesis | 0.0787 | Amino acid metabolism | Metabolism |
| | Phenylalanine, tyrosine and tryptophan biosynthesis | 0.2452 | Amino acid metabolism | Metabolism |
| | Tyrosine metabolism | 0.2452 | Amino acid metabolism | Metabolism |
| | Phenylalanine metabolism | 0.1847 | Amino acid metabolism | Metabolism |
| | Glycosaminoglycan degradation | 0.0787 | Glycan biosynthesis and metabolism | Metabolism |
| | Ubiquinone and other terpenoid-quinone biosynthesis | 0.2452 | Metabolism of cofactors and vitamins | Metabolism |
| | Porphyrin and chlorophyll metabolism | 0.2452 | Metabolism of cofactors and vitamins | Metabolism |
| | Nicotinate and nicotinamide metabolism | 0.0787 | Metabolism of cofactors and vitamins | Metabolism |
| | Zeatin biosynthesis | 0.0787 | Metabolism of terpenoids and polyketides | Metabolism |
| | Tropane, piperidine and pyridine alkaloid biosynthesis | 0.8660 | Biosynthesis of other secondary metabolites | Metabolism |
| | Isoquinoline alkaloid biosynthesis | 0.2452 | Biosynthesis of other secondary metabolites | Metabolism |
| | Protein processing in endoplasmic reticulum | 4.1924 | Folding, sorting and degradation | Genetic information processing |
| | ABC transporters | 0.1847 | Membrane transport | Environmental information processing |
| | Plant hormone signal transduction | 0.0787 | Signal transduction | Environmental information processing |
| | Endocytosis | 0.2452 | Transport and catabolism | Cellular processes |
| | Peroxisome | 0.1684 | Transport and catabolism | Cellular processes |
| S1 (S1 vs. S2) | Fatty acid metabolism | 0.3312 | Global and overview maps | Metabolism |
| | Metabolic pathways | 0.2067 | Global and overview maps | Metabolism |
| | Biosynthesis of secondary metabolites | 0.1584 | Global and overview maps | Metabolism |
| | Galactose metabolism | 0.2021 | Carbohydrate metabolism | Metabolism |
| | Ascorbate and aldarate metabolism | 0.2021 | Carbohydrate metabolism | Metabolism |
| | Pentose and glucuronate interconversions | 0.1584 | Carbohydrate metabolism | Metabolism |
| | Starch and sucrose metabolism | 0.1508 | Carbohydrate metabolism | Metabolism |
| | Amino sugar and nucleotide sugar metabolism | 0.0808 | Carbohydrate metabolism | Metabolism |

| | | | |
|---|---|---|---|
| Pentose phosphate pathway | 0.0618 | Carbohydrate metabolism | Metabolism |
| Photosynthesis - antenna proteins | 6.1531 | Energy metabolism | Metabolism |
| Photosynthesis | 3.3883 | Energy metabolism | Metabolism |
| Nitrogen metabolism | 0.1508 | Energy metabolism | Metabolism |
| Fatty acid elongation | 3.3883 | Lipid metabolism | Metabolism |
| Cutin, suberine and wax biosynthesis | 0.9580 | Lipid metabolism | Metabolism |
| Fatty acid biosynthesis | 0.2021 | Lipid metabolism | Metabolism |
| Biosynthesis of unsaturated fatty acids | 0.1508 | Lipid metabolism | Metabolism |
| Valine, leucine and isoleucine biosynthesis | 0.0618 | Amino acid metabolism | Metabolism |
| Phenylalanine metabolism | 0.1584 | Amino acid metabolism | Metabolism |
| Phenylalanine, tyrosine and tryptophan biosynthesis | 0.0618 | Amino acid metabolism | Metabolism |
| Arginine and proline metabolism | 0.0618 | Amino acid metabolism | Metabolism |
| Cyanoamino acid metabolism | 0.1508 | Metabolism of other amino acids | Metabolism |
| beta-Alanine metabolism | 0.0618 | Metabolism of other amino acids | Metabolism |
| Porphyrin and chlorophyll metabolism | 0.2021 | Metabolism of cofactors and vitamins | Metabolism |
| Limonene and pinene degradation | 0.1508 | Metabolism of terpenoids and polyketides | Metabolism |
| Brassinosteroid biosynthesis | 0.0618 | Metabolism of terpenoids and polyketides | Metabolism |
| Phenylpropanoid biosynthesis | 0.9580 | Biosynthesis of other secondary metabolites | Metabolism |
| Isoquinoline alkaloid biosynthesis | 0.0808 | Biosynthesis of other secondary metabolites | Metabolism |
| Stilbenoid, diarylheptanoid and gingerol biosynthesis | 0.0618 | Biosynthesis of other secondary metabolites | Metabolism |
| Flavonoid biosynthesis | 0.0618 | Biosynthesis of other secondary metabolites | Metabolism |
| Plant hormone signal transduction | 0.2067 | Signal transduction | Environmental information processing |

The pathways are grouped according to KEGG pathway maps (http://www.genome.jp/kegg/pathway.html). The pathway-enrichment analysis was significant with FDR < 0.005. K1: ‘Kiyomi’ at first stage; K2: ‘Kiyomi’ at second stage; S1: ‘Shiranui’ at first stage; S2: ‘Shiranui’ at second stage.

## Table S3 The genes involved in pectin metabolism

| Gene | Gene expression levels | | | | | Contigs ID |
|---|---|---|---|---|---|---|
| | K1 | S1 | K2 | S2 | Average | |
| GAUT (α-1,4-D-galacturonosyltransferase) | | | | | | |
| GAUT8 | 8.948 | 13.160 | 23.602 | 23.337 | 17.261 | orange1.1g008561m.g |
| GAUT9 | 6.666 | 14.830 | 15.204 | 24.885 | 15.396 | orange1.1g034721m.g |
| GAUT4 | 10.790 | 7.408 | 24.553 | 18.060 | 15.203 | orange1.1g005035m.g |
| GAUT15 | 7.433 | 7.528 | 8.930 | 11.478 | 8.842 | orange1.1g009468m.g |
| GAUT1 | 1.591 | 2.392 | 6.531 | 5.739 | 4.063 | orange1.1g005683m.g |
| GAUT7 | 2.405 | 3.621 | 4.466 | 4.620 | 3.778 | orange1.1g006506m.g |
| GATL (GAUT-like) | | | | | | |
| GATL3 | 4.082 | 7.028 | 21.453 | 33.278 | 16.460 | orange1.1g042515m.g |
| GATL10 | 5.113 | 6.207 | 7.445 | 38.788 | 14.388 | orange1.1g023261m.g |
| GATL9 | 5.600 | 7.454 | 14.934 | 26.867 | 13.714 | orange1.1g047424m.g |
| GATL7 | 1.356 | 1.189 | 13.853 | 10.995 | 6.848 | orange1.1g017643m.g |
| PME (Pectin methylesterase) | | | | | | |
| PME31 | 360.666 | 265.745 | 249.429 | 229.491 | 276.333 | orange1.1g021162m.g |
| PME3 | 56.331 | 134.925 | 179.135 | 229.123 | 149.878 | orange1.1g008261m.g |
| PME1 | 93.437 | 104.005 | 69.602 | 82.923 | 87.491 | orange1.1g007830m.g |
| PME61 | 6.760 | 11.931 | 100.757 | 89.896 | 52.336 | orange1.1g017225m.g |
| PME40 | 4.841 | 5.739 | 53.799 | 32.092 | 24.117 | orange1.1g015794m.g |
| PME12 | 2.809 | 2.343 | 37.975 | 42.902 | 21.507 | orange1.1g044734m.g |
| PME18 | 14.888 | 7.804 | 17.930 | 18.825 | 14.862 | orange1.1g008722m.g |
| PME32 | 4.846 | 15.279 | 18.484 | 14.359 | 13.242 | orange1.1g010718m.g |
| PME21 | 0.045 | 36.388 | 0.049 | 10.456 | 11.734 | orange1.1g039111m.g |
| PME51 | 2.498 | 3.416 | 13.995 | 10.313 | 7.555 | orange1.1g009085m.g |
| PME54 | 0.046 | 1.343 | 7.468 | 19.630 | 7.122 | orange1.1g009418m.g |
| PMEI (PME inhibitor) | | | | | | |
| | 163.747 | 565.485 | 608.092 | 1411.688 | 687.253 | orange1.1g029436m.g |
| | 934.878 | 808.492 | 95.787 | 215.350 | 513.627 | orange1.1g038081m.g |
| | 16.625 | 41.428 | 67.400 | 120.620 | 61.518 | orange1.1g027094m.g |
| | 71.714 | 103.412 | 33.365 | 23.903 | 58.098 | orange1.1g031799m.g |
| | 89.300 | 35.788 | 49.355 | 40.896 | 53.835 | orange1.1g037982m.g |
| | 26.002 | 23.973 | 48.052 | 49.829 | 36.964 | orange1.1g026791m.g |
| | 13.600 | 7.628 | 18.318 | 6.540 | 11.522 | orange1.1g029211m.g |
| | 0.719 | 2.564 | 19.448 | 6.856 | 7.397 | orange1.1g028183m.g |
| | 5.601 | 0.288 | 12.779 | 3.746 | 5.603 | orange1.1g040679m.g |
| PAE (pectin acetylesterase) | | | | | | |
| PAE8 | 144.567 | 105.868 | 102.675 | 78.796 | 107.976 | orange1.1g013734m.g |
| PAE2 | 21.930 | 20.612 | 21.044 | 20.727 | 21.078 | orange1.1g026496m.g |

| | | | | | | |
|---|---|---|---|---|---|---|
| PAE6 | 6.394 | 13.686 | 21.871 | 19.165 | 15.279 | orange1.1g014548m.g |
| Pectate lyase /Pectin lyase | | | | | | |
| | 161.484 | 240.564 | 441.108 | 450.867 | 323.505 | orange1.1g015702m.g |
| | 426.901 | 460.503 | 199.835 | 115.311 | 300.637 | orange1.1g015569m.g |
| | 136.012 | 351.736 | 110.389 | 125.756 | 180.973 | orange1.1g010437m.g |
| | 181.484 | 137.324 | 128.988 | 111.794 | 139.897 | orange1.1g013402m.g |
| | 0.492 | 0.467 | 156.139 | 95.815 | 63.228 | orange1.1g012057m.g |
| | 47.112 | 61.330 | 34.023 | 44.671 | 46.784 | orange1.1g038762m.g |
| | 15.615 | 18.191 | 50.206 | 55.748 | 34.940 | orange1.1g011204m.g |
| | 1.277 | 2.246 | 46.330 | 42.612 | 23.116 | orange1.1g009075m.g |
| | 39.103 | 35.315 | 3.151 | 7.273 | 21.211 | orange1.1g039631m.g |
| | 12.561 | 14.578 | 16.818 | 17.674 | 15.408 | orange1.1g020121m.g |
| | 9.502 | 17.828 | 15.139 | 17.566 | 15.008 | orange1.1g021449m.g |
| | 2.875 | 2.739 | 13.482 | 13.031 | 8.031 | orange1.1g009748m.g |

Genes with expression levels greater than 10, except for *GAUT1* and *GAUT7*, are shown. The average expression levels are from the two citrus cultivars at two developmental stages. The genes/contigs are listed in the order of the average expression levels. K1: ‘Kiyomi’ at first stage; K2: ‘Kiyomi’ at second stage; S1: ‘Shiranui’ at first stage; S2: ‘Shiranui’ at second stage.

**Table S4 The genes involved in cellulose metabolism**

| Gene | Gene expression levels | | | | | Contigs ID |
|---|---|---|---|---|---|---|
| | K1 | S1 | K2 | S2 | Average | |
| CESA (cellulose synthase) | | | | | | |
| CESA9 | 4.011 | 18.808 | 66.307 | 114.029 | 50.788 | orange1.1g001373m.g |
| CESA3 | 11.205 | 21.171 | 49.690 | 49.374 | 32.860 | orange1.1g001413m.g |
| CESA1 | 9.561 | 28.445 | 37.000 | 42.463 | 29.367 | orange1.1g001399m.g |
| CESA6 | 3.057 | 6.938 | 15.380 | 15.247 | 10.155 | orange1.1g001369m.g |
| CESA8 | 0.017 | 0.171 | 0.396 | 0.237 | 0.205 | orange1.1g002020m.g |
| CESA7 | 0.006 | 0.005 | 0.405 | 0.298 | 0.178 | orange1.1g039060m.g |
| CESA4 | 0.012 | 0.032 | 0.242 | 0.182 | 0.117 | orange1.1g001574m.g |
| SUS (sucrose synthase) | | | | | | |
| SUS3 | 120.941 | 168.212 | 74.538 | 113.362 | 119.263 | orange1.1g003492m.g |
| SUS4 | 40.360 | 52.039 | 91.822 | 88.906 | 68.281 | orange1.1g003661m.g |
| SUS6 | 1.794 | 1.366 | 1.995 | 1.536 | 1.673 | orange1.1g002909m.g |
| SUS2 | 0.000 | 0.060 | 0.077 | 0.112 | 0.062 | orange1.1g003726m.g |
| CEL (cellulase) | | | | | | |
| CEL1 | 0.695 | 1.123 | 7.052 | 10.352 | 4.805 | orange1.1g012581m.g |
| CEL3 | 0.765 | 2.988 | 0.765 | 1.676 | 1.548 | orange1.1g015354m.g |
| CEL2 | 0.040 | 0.030 | 0.012 | 0.017 | 0.025 | orange1.1g010632m.g |

The average expression levels are from the two citrus cultivars at two developmental stages. The genes/contigs are listed in the order of the average expression levels. K1: ‘Kiyomi’ at first stage; K2: ‘Kiyomi’ at second stage; S1: ‘Shiranui’ at first stage; S2: ‘Shiranui’ at second stage.

**Table S5 The genes involved in hemicellulose (xyloglucans and xylan) metabolism**

| Gene | Gene expression levels | | | | | Contigs ID |
|---|---|---|---|---|---|---|
| | K1 | S1 | K2 | S2 | Average | |
| CSLC (cellulose synthase-like C) | | | | | | |
| CSLC4 | 1.433 | 1.256 | 4.256 | 3.291 | 2.559 | orange1.1g006104m.g |
| CSLC5 | 2.879 | 1.442 | 5.915 | 2.906 | 3.285 | orange1.1g005507m.g |
| CSLC12 | 1.973 | 11.843 | 16.548 | 46.068 | 19.108 | orange1.1g005700m.g |
| XXT (xyloglucan xylosyltransferase) | | | | | | |
| XXT1 | 14.899 | 17.675 | 20.451 | 21.754 | 18.695 | orange1.1g012077m.g |
| XXT2 | 14.211 | 17.591 | 46.982 | 52.042 | 32.706 | orange1.1g047753m.g |
| XXT3 | 7.564 | 7.522 | 8.641 | 10.988 | 8.679 | orange1.1g012705m.g |
| MUR | | | | | | |
| MUR2 | 3.027 | 2.781 | 10.670 | 8.840 | 6.329 | orange1.1g039620m.g |
| MUR3 | 3.142 | 4.516 | 9.263 | 23.693 | 10.153 | orange1.1g007197m.g |
| XLT (xyloglucan galactosyltransferase) | | | | | | |
| XLT2 | 16.916 | 18.515 | 85.522 | 80.970 | 50.481 | orange1.1g011220m.g |
| AXY (acetyltransferase) | | | | | | |
| AXY4 | 0.879 | 0.463 | 2.476 | 1.837 | 1.414 | orange1.1g013153m.g |
| AXY4L (AXY4 Like) | 0.585 | 0.583 | 2.345 | 1.548 | 1.265 | orange1.1g014612m.g |
| BGAL (Beta galactosidase) | | | | | | |
| BGAL10 | 2.612 | 3.191 | 3.203 | 3.362 | 3.092 | orange1.1g004525m.g |
| XYL (xylosidase ) | | | | | | |
| XYL1 | 4.737 | 4.792 | 27.915 | 19.161 | 14.151 | orange1.1g002256m.g |
| IRX (irregular xylem phenotype) | | | | | | |
| IRX7 | 60.792 | 61.008 | 55.310 | 71.390 | 62.125 | orange1.1g039568m.g |
| IRX8/GAUT12 | 7.746 | 5.306 | 6.650 | 4.645 | 6.086 | orange1.1g007529m.g |
| IRX9-L (IRX9-Like) | 6.963 | 10.757 | 15.223 | 15.339 | 12.070 | orange1.1g016070m.g |
| IRX10 | 1.679 | 2.085 | 2.069 | 1.972 | 1.951 | orange1.1g038885m.g |
| IRX10-L (IRX10-Like) | 6.330 | 9.254 | 7.262 | 9.126 | 7.993 | orange1.1g048016m.g |
| IRX14-L (IRX14-Like) | 10.713 | 16.027 | 14.428 | 13.478 | 13.661 | orange1.1g010452m.g |
| IRX15-L (IRX15-Like) | 0.000 | 0.350 | 0.074 | 0.190 | 0.153 | orange1.1g021017m.g |

The average expression levels are from the two citrus cultivars at two developmental stages. The genes/contigs are listed in the order of the gene names. *MUR2*, also known as *FUT1*, encodes a fucosyl transferase. *MUR3* encodes a xyloglucan galactosyltransferase specific for

transferring the galactosyl residue to the third xylosyl residue (Madson *et al.*, 2003*). K1: ‘Kiyomi’ at first stage; K2: ‘Kiyomi’ at second stage; S1: ‘Shiranui’ at first stage; S2: ‘Shiranui’ at second stage.

*Madson M, Dunand C, Li X, Verma R, Vanzin GF, Caplan J, Shoue DA, Carpita NC, Reiter WD. The MUR3 gene of Arabidopsis encodes a xyloglucan galactosyltransferase that is evolutionarily related to animal exostosins. Plant Cell. 2003;15:1662–1670.
